# Polymerization mechanism of natural lacquer sap with special phase structure

**DOI:** 10.1038/s41598-020-69823-0

**Published:** 2020-07-30

**Authors:** Jianhong Yang, Nan Chen, Jianfeng Zhu, Jun Cai, Jianping Deng, Feifei Pan, Lianghe Gao, Zhenfei Jiang, Fengqin Shen

**Affiliations:** 1 School of Environmental and Safety Engineering, Changzhou University, Changzhou, 213164 China; 2 Key Laboratory of Fermentation Engineering (Ministry of Education), Hubei Key Laboratory of Industrial Microbiology, Hubei University of Technology, Wuhan, 430068 China; 3 Changzhou Liu Guojun Vocational Technology College, Changzhou, 213025 Jiangsu China

**Keywords:** Plant sciences, Chemistry, Materials science

## Abstract

Lacquer sap is a water-in-oil natural emulsion with high viscosity. In nature, it exudes from the phloem of lacquer tree to repair its wounds in the presence of O_2_. So far, it is unclear how rapid and smooth polymerization of urushiol is achieved in such a viscous sap. Here, we find that there is a diffuse interface layer with 2.43 nm of thickness between two phases. The interface layer consists of urushiol, urushiol–laccase complex, urushiol–stellacyanin complex and water-insoluble glycoprotein. Polymerization of urushiol is realized by multicomponent synergistic effect. Radicals are first formed by laccase-catalyzed oxidation of urushiol at the interface layer, then are transferred to the urushiol oil phase via wate-insoluble glycoprotein and initiate the polymerization of urushiol there. Stellacyanin inhibits the formation of certain radicals and controls the concentration of phenoxy radicals at the interface layer. Through the inhibition of radicals by stellacyanin and the electron transfer mediated by water-insoluble glycoprotein, the polymerization of urushiol at the interface layer is inhibited. This ensures that O_2_ can continuously penetrate into the aqueous phase to oxidize the reduced laccase so that the urushiol polymerization can continue smoothly. This polymerization mechanism provides an idea for developing new chemical reaction systems.

## Introduction

Today, we can enjoy some exquisite lacquerwares in museums all over the world. Although some have been preserved for thousands of years, they still show their long-lasting beauty. Their surface coatings were made of a sap from lacquer tree growing in Asia. When the bark of lacquer tree is injured, a viscous sap exudes from its phloem and then forms a hard film to cover the wound in the presence of oxygen^1^. The lacquer sap obtained from *Rhus vernicifera* lacquer tree mainly consists of urushiol (60–65%), water (20–30%), lacquer polysaccharide (3–7%), water-insoluble glycoprotein (~ 1–2%), laccase (~ 0.2%) and stellacyanin (~ 0.02%)^2–4^. In China, Japan and other Asian countries, it has been used as a coating material for several thousand years^5,6^. Unlike the drying of traditional synthetic coatings by solvent evaporation, lacquer film, which is a highly cross-linked three-dimensional polymer, is formed by the oxidation polymerization of urushiol monomer catalyzed by laccase under high humidity^2,7–11^. The function of water is to maintain laccase activity and lacquer polysaccharide plays a role in preventing the fast loss of water^12^. So far, however, the understanding of the polymerization mechanism of lacquer sap is nothing more than that.

The bottleneck of further exploring the polymerization mechanism of lacquer sap was the lack of understanding of its phase structure. It is generally considered that the sap is a W/O (water-in-oil) emulsion with urushiol as a continuous phase and water as a dispersed phase^9^. Water-insoluble glycoprotein could be an emulsifier^9,11^. Kumanotani believed that laccase could exist in both phases^2,13^. The convincing evidences were not provided to support these statements. In this paper, we aim to construct the phase interface structure and polymerization mechanism of lacquer sap.

## Results and discussions

**Figure 1:**
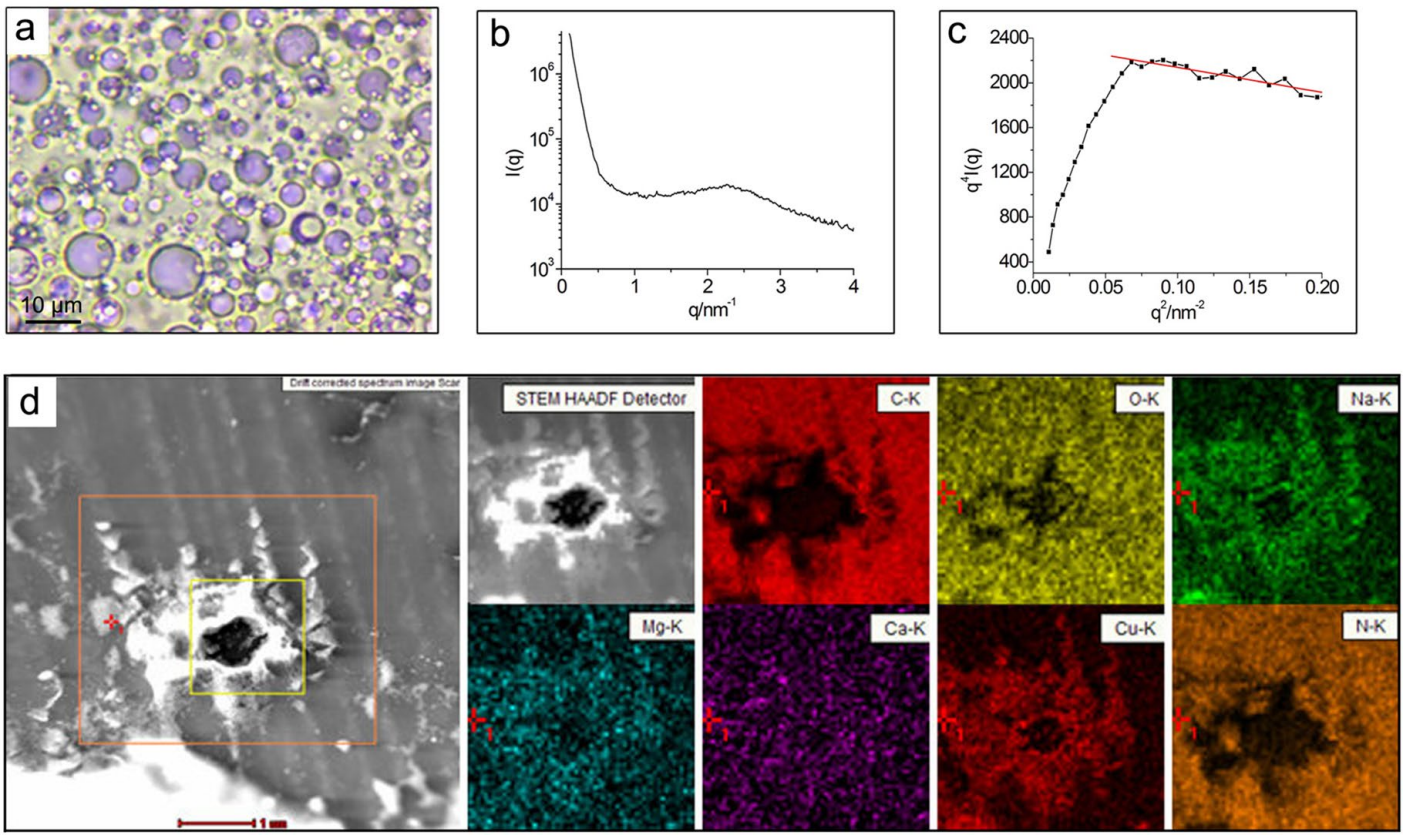
Characterization of phase structure of lacquer sap. (**a**) Biological microscope photograph of lacquer sap (× 1,000 times); (**b**) SAXS curve plotted as I(q) versus q for lacquer sap; (**c**) SAXS curve plotted as q^4^I(q) versus q^2^; (**d**) TEM-EDS images of the element distribution of the cured lacquer film.

A direct observation of lacquer sap was carried out using optical microscope. It could be seen from Fig. 1a that a lot of spherical water droplets were dispersed in the urushiol and their sizes were about 0.5–10 μm. This also showed that raw lacquer sap was a W/O emulsion. The same results could be also obtained from its Cryo-FESEM image and the SEM image of its dry film (Supplementary Fig. S1). Raw lacquer sap was also directly analyzed by SAXS. Figure 1b shows its scattering curve. There was a single broad scattering peak on the scattering curve. This was a typical scattering curve of two-phase system^14^. In the SAXS curve plotted as *q*^4^*I*(*q*) ~ *q*^2^ (Fig. 1c), it showed a linear relationship with a negative slope in the region of 0.21 nm^−2^ > *q*^2^ > 0.06 nm ^−2^. Meanwhile, the value of the interface thickness parameter σ was 0.97 nm according to the method in the literature^15,16^ These are the characteristics of a non-ideal two-phase system with a diffuse interface layer^15,16^. Its average phase interface thickness (ΔR) was 2.43 nm according to the equation: ΔR = (2π)^1/2^*σ*^15,16^.

Further, we located each component in lacquer sap. A FITC-labelled laccase was dispersed into the lacquer sap and the punctate distribution of green fluorescence was seen (Supplementary Fig. S2), indicating that laccase existed in water. In the cured lacquer film, Cu element was found to only exist in the location of the cave caused by water evaporation (Fig. 1d). Since *Rhus vernicifera* laccase is a multicopper glycoprotein and stellacyanin is a single-copper glycoprotein^2,8,17–22^, they should exist in the water. In addition, it was also found from Fig. 1a that water droplets were bluish violet and a clear circle layer of dark bluish violet was located at the two-phase interface. It is known that the aqueous solutions of stellacyanin and laccase absorb the light of ~ 604 and ~ 614 nm, respectively^17–20^. Furthermore, the color at the two-phase interface was darker than that inside the water droplet. These indicated that stellacyanin and laccase mainly existed in the junction of two phases.

In the cured lacquer film, Na, Mg and Ca elements only existed in the location of the cave (Fig. 1d). Na, Mg and Ca elements were found to bind to water-soluble lacquer polysaccharide^4,23–26^ so polysaccharide should exist in water. The N element was found around the cave, which could be related to laccase and stellacyanin. However, the N element also existed in the lacquer film away from the cave (Fig. 1d). The results from Cryo-FESEM-EDS analysis also showed that there was N element in the oil phase (Supplementary Fig. S3). This should result from water-insoluble glycoprotein. Besides, lacquer sap was divided into two layers under high-speed centrifugation. The upper layer was urushiol and the bottom layer was still an emulsion (Supplementary Fig. S4). The content of water-insoluble glycoprotein was found to be 0.96 wt% in the upper layer and 1.61 wt% in the bottom layer, suggesting that water-insoluble glycoprotein was present both in the urushiol phase and at the interface layer.

Considering the amphiphilic properties of urushiol, it could form the phase interface layer of lacquer sap. However, the emulsion obtained by dispersing water directly into urushiol was unstable and the phase separation was observed within 24 h (Supplementary Fig. S5). Obviously, urushiol was not sufficient to form a stable interface layer alone due to its low hydrophilic–lipophilic balance value (< 0.8)^27^. If laccase was added to the urushiol/H_2_O system, the emulsion could be stable in 6 days (Supplementary Fig. S6). Because urushiol is the substrate of laccase, it was reasonably thought that a laccase-urushiol complex acted as an emulsifier to form the phase interface layer, as observed in Fig. 1a. Similarly, the urushiol/H_2_O emulsion containing stellacyanin was also more stable than the urushiol/H_2_O system (Supplementary Fig. S7). It was inferred that the stellacyanin–urushiol complex was also involved in the formation of the interface layer. However, the stability of the emulsion was lower than that with the laccase. In addition, for the urushiol/H_2_O emulsions containing laccase, stellacyanin and lacquer polysaccharide, the phase separation occurred rapidly within 24 h (Supplementary Fig. S5). A large number of carboxyl anions and metal cations on lacquer polysaccharide could be responsible for the instability of the emulsion^4,25^. Evidently, the urushiol/H_2_O emulsion containing only the above components was not stable enough.

**Figure 2:**
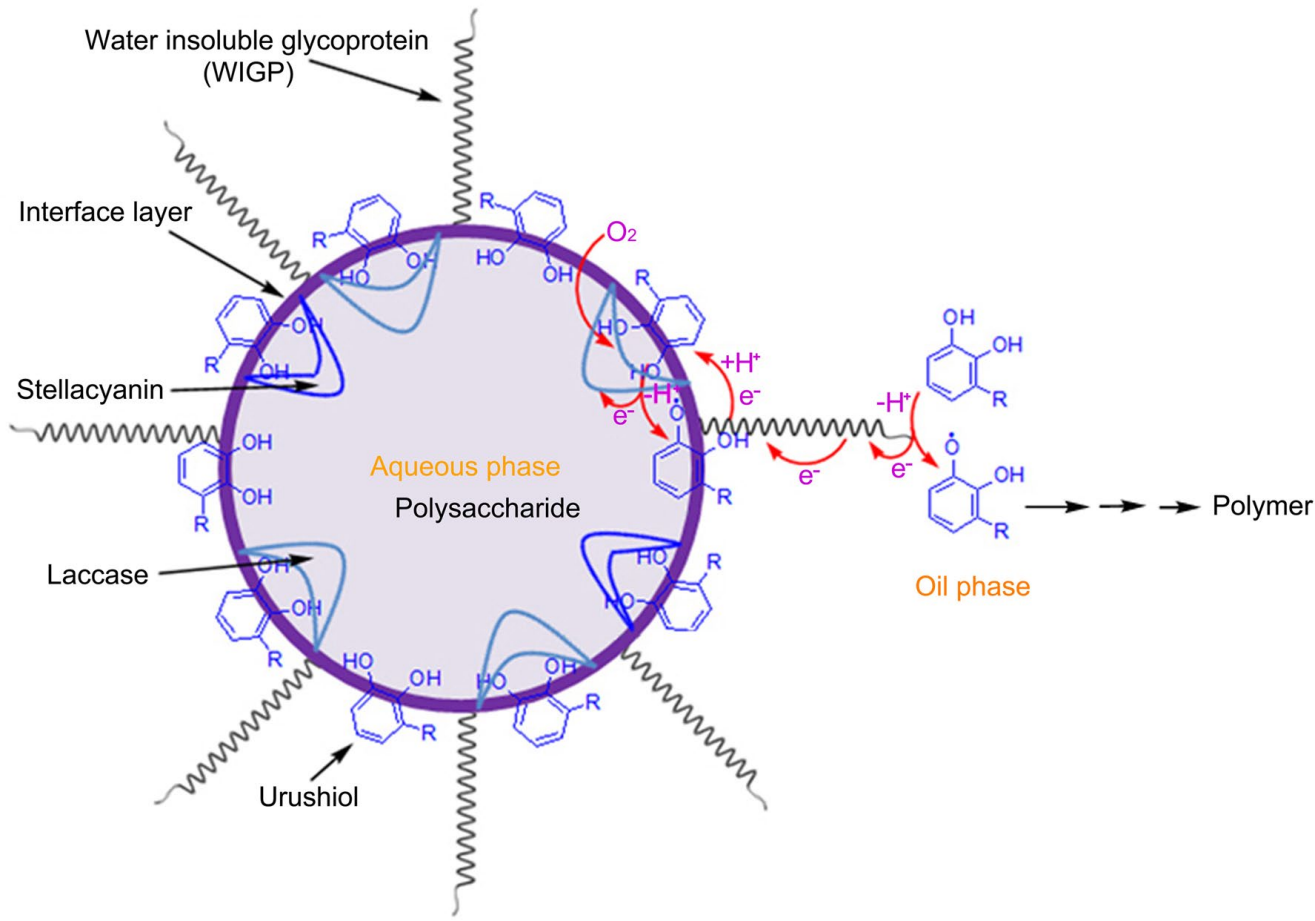
Schematic illustration of the phase interface structure of lacquer sap and its drying mechanism by long-range electron transfer via water-insoluble glycoprotein between oil phase and aqueous phase.

Furthermore, we investigated the effect of water-insoluble glycoprotein on the stability of the W/O emulsion. Since isolated water-insoluble glycoprotein was insoluble in urushiol, an urushiol with water-insoluble glycoprotein (UGP) obtained by the centrifugation was used for the study (See Supplementary Fig. S3). Its content of water-insoluble glycoprotein (0.96 wt%) was lower than that in the sap (1.39 wt%). However, like the lacquer sap, both UGP/H_2_O emulsion and UGP/H_2_O emulsion with laccase showed high stability. No emulsion delamination was observed at room temperature for more than 3 months (Supplementary Fig. S8). It was thus clear that water-insoluble glycoprotein played a crucial role in stabilizing the W/O emulsion. Like many protein-stabilised emulsions^28^, water-insoluble glycoprotein could absorb at the oil–water interface to provide stability to the emulsion. Based on the above results, the phase interface layer of lacquer sap could consist of urushiol, laccase–urushiol complex, stellacyanin–urushiol complex and water-insoluble glycoprotein. Raw lacquer sap can be stored steadily for several years due to the action of the specific phase interface layer. The possible phase structure is shown in Fig. 2 and Supplementary Fig. S9.

For the drying of lacquer sap, urushiol is first oxidized by laccase in the presence of O_2_ to give a phenolic oxygen free radical. The radical then rearranges to form a semiquinone radical and reacts rapidly with a neighboring urushiol molecule to produce a biphenyl dimer. The dimers further polymerize to form the polymer^13^. When lacquer sap (LS) covered the wound of the lac tree or was applied on the substrate, it was not stirred during its drying. Meanwhile, the sap was very sticky and its viscosity was more than 7,000 mPa s at 20 °C (Table 1), so the diffusion of these radicals formed in the phase interface into the interior of the urushiol phase should be slow. It seemed that the lacquer sap should dry slowly. However, a lacquer sap with a short storage period could dry rapidly. A lacquer sap with 4-year storage (LS4) took longer time to achieve the hard dryness due to the inactivation of some laccase. When it was mixed with active laccase, its hard dryness time was greatly shortened (Table 1). It was also observed that the urushiol/H_2_O emulsions with laccase (UHEL) could dry quickly. When its laccase content was reduced from 0.24 to 0.12 wt%, the hard dryness time of the emulsion was greatly prolonged (Table 1). Moreover, the viscosity of lacquer sap was ~ 50 times higher than that of UHEL (Table 1). If the polymerization of the urushiol was initiated by the diffusion of free radicals into the oil phase, lacquer sap should dry much more slowly than UHEL. Obviously, it was difficult to explain the rapid drying of lacquer sap by the free radical diffusion.

**Table 1:** The viscosities and drying properties of lacquer sap and some prepared urushiol emulsions.

Sample^a^	Additive	Ratio (wt%)	Viscosity^b^ (mPa s)	Temp (^o^C)	RH^c^ (%)	Drying time (h:min)	
						TD^d^	HD^e^
LS	–	–	7,635 ± 13	30	80	3:50	12:20
LS4	–	–	7,069 ± 10	30	80	29:50	65:55
	Laccase	0.24%	–	30	80	0:25	1:45
UGP	–	–	874.1 ± 0.9	30	80	NC	NC^f^
UGPH	–	–	2,050 ± 9	30	80	NC	NC
UGPHL-1	Laccase	0.12%	2,197 ± 11	30	80	4:45	13:40
UGPHL-2	Laccase	0.24%	2,244 ± 12	30	80	3:20	10:30
Urushiol	–	–	52.5 ± 1.0	30	80	NC	NC
UHEL-1	Laccase	0.12%	124.6 ± 0.30	30	80	17:10	29:30^g^
UHEL-2	Laccase	0.24%	144.4 ± 0.31	30	80	4:30	9:10^g^

*LS* lacquer sap; *LS4* lacquer sap has stored for 4 years; *UGP* urushiol with 0.96 wt% of water-insoluble glycoprotein; *UGPH* UGP/H_2_O emulsion; *UGPHL-1 and UGPHL-2* UGP/H_2_O emulsion with 0.12 wt% and 0.24 wt% of laccase, respectively; *UHEL-1 and UHEL-2* urushiol/H_2_O emulsion with 0.12 wt% and 0.24 wt% of laccase, respectively.

^a^The content of water in all samples was controlled at 21 wt%.

^b^Results were expressed as means ± S.D. (n = 5).

^c^Relative humidity.

^d^Touch dryness.

^e^Hard dryness.

^f^Do not cure within 40 days.

^g^The time of partial drying of the sample. There was also some uncured urushiol regions on the sample film.

Further, it was also found that the cured films from the lacquer sap dried better than those from UHEL. Uncured urushiol region was not observed on the films of lacquer sap (Supplementary Fig. S10). However, there were many spot-like uncured urushiol regions on the film of UHEL with 0.24 wt% of laccase. Especially, large uncured regions could be clearly observed in the film from the urushiol/H_2_O emulsion with 0.12 wt% of laccase even though it had been dried for 48 h (Supplementary Fig. S10). Therefore, we believed that there was a substance that acted as a bridge between two phases in the lacquer sap. It could play the role of long range mediated electron transfer. It is well known that electron transfer processes in peptides and proteins are fundamental for living organisms^29,30^. For the lacquer sap from lacquer tree, this substance could be water-insoluble glycoprotein.

In order to confirm the effect of water-insoluble glycoprotein, the drying properties of UGP/H_2_O emulsions with laccase (UGPHL) were investigated. Two UGPHL samples containing different laccase levels (0.24 wt% and 0.12 wt%) showed a cured time comparable to that of raw lacquer sap (Table 1). Compared with UHELs, their viscosity was much higher, but their cured time was close to that of UHEL with 0.24 wt% laccase and much shorter than that of UHEL with 0.12 wt% laccase. Unlike UHELs, as the content of laccase decreased from 0.24 to 0.12 wt%, their cured time did not increase significantly. In addition, their cured films were almost the same as the raw lacquer film (Supplementary Fig. S10). These further suggested that water-insoluble glycoprotein could mediate the electron transfer. Because of the presence of water-insoluble glycoprotein in urushiol phase and phase interfaces, it mediated the electron transfer between two phases and in the urushiol phase, accelerating the drying of lacquer sap. Based on the above conclusions, a credible polymerization mechanism of urushiol in lacquer sap is shown in Fig. 2.

**Figure 3:**
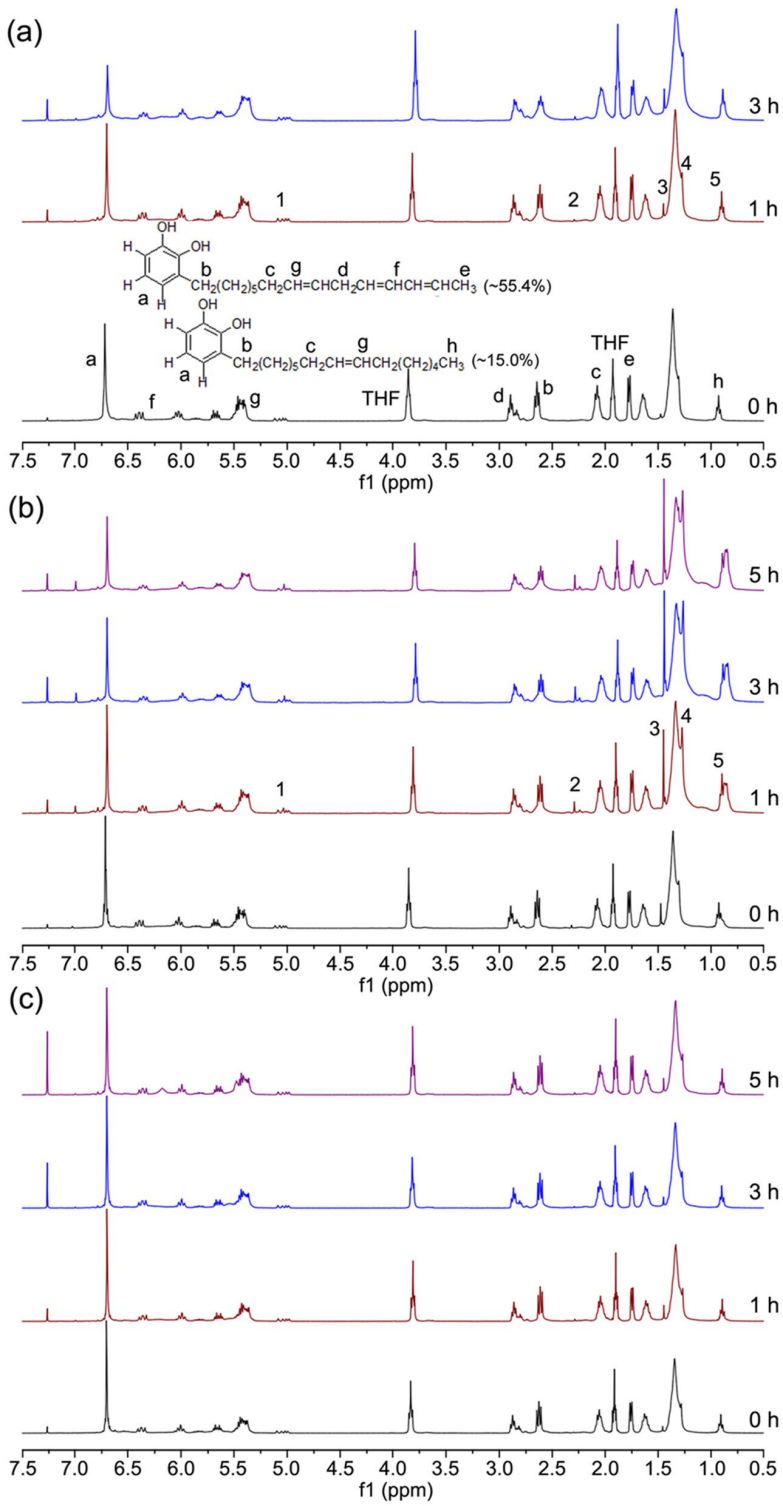
Inhibitory effects of stellacyanin on the polymerization of urushiol in the drying process. (**a**) ^1^HNMR spectra of THF extract after raw lacquer sap drying for 0 h,1 h and 3 h, respectively; (**b**) ^1^H NMR spectrum of THF extract after urushiol/H_2_O emulsion with active laccase (0.24 wt%) drying for 0 h, 1 h, 3 h and 5 h; the intensity of the peaks 1–5 rapidly increased compared with those in lacquer sap; (**c**) ^1^H NMR spectrum of THF extract after urushiol/H_2_O emulsion with active laccase (0.24 wt%) and stellacyanin (0.024 wt%) drying for 0 h, 1 h, 3 h and 5 h. This ^1^H NMR spectrum was similar to that of raw lacquer sap.

The role of stellacyanin was also further investigated in lacquer sap. We used NMR to track the drying process of lacquer sap, UHEL and UGPHL. It could be found that the drying mechanism of lacquer sap was not exactly the same as that of UHEL and UGPHL by comparing their ^1^H NMR spectra (Fig. 3 and Supplementary Figs. S11, S12). The peaks **1**–**5** at ~ 5.03 ppm, ~ 2.28 ppm, ~ 1.44 ppm, ~ 1.26 ppm and ~ 0.86 ppm were related to the hydrogen atoms on the alkyl chain of urushiol. In the ^1^H NMR spectra of UHEL (Fig. 3b) and UGPHL (Supplementary Fig. S11), as the drying time increased, the intensity of these peaks rapidly increased, but it was not for the raw lacquer sap (Fig. 3a). These indicated that some reactions had already occurred on the alkyl chain of urushiol, but the same reactions were not clearly observed in the lacquer sap. When stellacyanin was added into UHEL and UGPHL, like raw lacquer sap, the intensity of these peaks did not increase significantly during the drying process (Fig. 3c and Supplementary Fig. S12). At the same time, the addition of stellacyanin also slowed down the decreasing rate of the protons on phenyl ring (~ 6.71 ppm), =C–CH_2_–C= (~ 2.89 ppm), Ar–CH_2_–R (~ 2.64 ppm), R–CH_2_–C=C– (~ 2.07 ppm) and –C=C–CH_3_ (~ 1.77 ppm). Obviously, stellacyanin inhibited certain radical polymerization, which involved the reactions both on the alkyl chain of urushiol and on the phenyl ring of urushiol. Since stellacyanin possessed a low oxidation–reduction potential of 184 mV and could act as a quinone/semiquinone reductase, its role was assumed to regulate the concentrations of aryloxy radicals and quinones involved in the laccase-catalysed oxidation of urushiol^31–33^. Thus, the effect of stellacyanin on laccase-catalyzed polymerization of urushiol could be due to its inhibition of free radical concentration.

**Figure 4:**
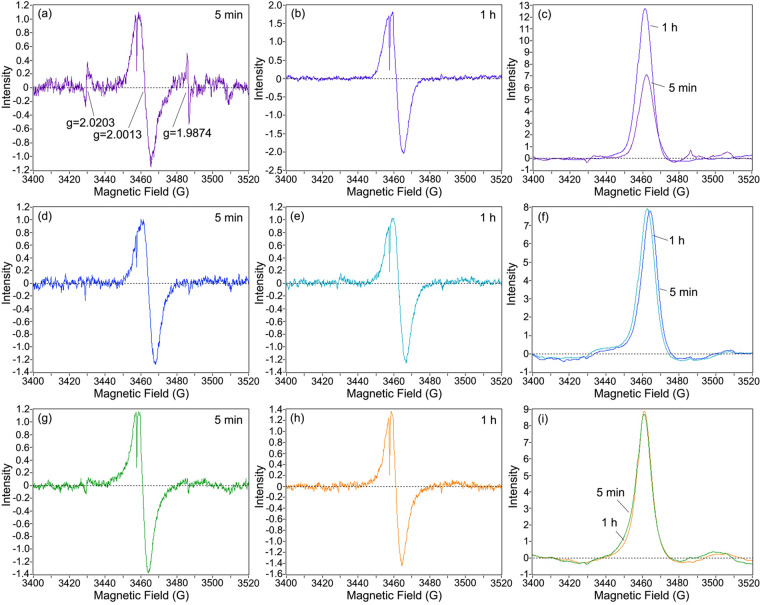
ESR spectra of samples in the drying process. ESR spectra of urushiol/H_2_O emulsion with laccase (0.24 wt%) (**a**, 5 min; **b**, 1 h) and their integrograms (**c**); ESR spectra of urushiol/H_2_O emulsion with laccase (0.24 wt%) and stellacyanin (0.024 wt%) (**d**, 5 min; **e**, 1 h) and their integrograms (**f**); ESR spectra of raw lacquer sap (**g**, 5 min; **h**, 1 h) and their integrograms (**i**).

Further, we used ESR to analyze the free radicals produced during the sample drying (Fig. 4). In the ESR spectra of the urushiol/H_2_O emulsion with laccase at 5 min, there were three signals, whose g values were 2.0203, 2.0013 and 1.9874, respectively (Fig. 4a). The broad signal at g = 2.0013 could be assigned to phenoxy radicals^34^. As the drying time increased to 1 h, its intensity greatly increased, indicating the increase of the concentration of phenoxy radicals (Fig. 4b,c). However, for the emulsion with stellacyanin and lacquer sap, the signal changed little with increase the time (Fig. 4d–i). This suggested that stellacyanin could control the concentration of aryloxy radicals, which was consistent with the hypothesis in the literature^32,33^. In addition, in the ESR spectra of the urushiol/H_2_O emulsion with laccase, when the drying time was 5 min, there were two signals at g = 2.0203 and 1.9874. The free radicals associated with the signals were unclear. As the drying time increased to 1 h, the two signals become very weak, indicating these radicals appeared mainly in the early stages of the polymerization process (Fig. 4b). In the ESR spectra of the emulsion with stellacyanin and lacquer sap, the two signals were very weak at 5 min (Fig. 4d,g). This indicated that some free radicals were also inhibited by stellacyanin.

Based on the ^1^H NMR spectra of UHEL, UGPHL and their samples modified with stellacyanin (Fig. 3b,c and Supplementary Figs. S11, S12), we could also find that the effects of stellacyanin on the decreasing rates of the protons on phenyl ring of urushiol were different with increasing the drying time. The decrease of the protons on the phenyl ring can be regarded as a sign of urushiol polymerization. For UHEL and UGPHL with 0.24 wt% of laccase, the protons on phenyl ring decreased by 47.1% and 59.1% after 3 h, respectively (Supplementary Table S1). However, when 0.024 wt% of stellacyanin was added into UHEL and UGPHL, their protons on the phenyl ring decreased by 18.1% and 47.2% after 3 h, respectively (Supplementary Table S1). Although stellacyanin inhibited the radical polymerization of urushiol, this inhibition effect on UHEL greatly exceeded that on UGPHL. We inferred that this difference was due to the presence or absence of water-insoluble glycoprotein. For UHEL, the free radicals formed at the interface layer entered the urushiol phase mainly by the diffusion. Stellacyanin inhibition of the free radical concentration necessarily caused low free radical diffusion rate and slow polymerization of urushiol. As for UGPHL, although its higher viscosity affected the diffusion of its free radicals, due to the presence of water-insoluble glycoprotein, the radicals could be rapidly transferred to the urushiol phase via water-insoluble glycoprotein. Thus, the inhibitory effect of stellacyanin was not significant. Likewise, for raw lacquer sap, it also showed rapid polymerization of urushiol due to the action of water-insoluble glycoprotein (Supplementary Table S1).

In addition, it is very important for the polymerization of urushiol to produce continuously the radicals by laccase. Due to the radical inhibition of stellacyanin and water-insoluble glycoprotein-mediated electron transfer, the concentration of free radicals in the interface layer was maintained at a low level and the polymerization of urushiol was inhibited at the interface layer, so that O_2_ could continuously penetrate to oxidize the reduced laccase in the drying process of lacquer sap. In this way, the radicals could be continuously produced by laccase and then transferred to the urushiol phase via water-insoluble glycoprotein to ensure the smooth polymerization of urushiol there.

## Conclusions

In summary, for the W/O type lacquer sap, its phase interface layer consisted of its several components such as urushiol, laccase, stellacyanin and water-insoluble glycoprotein. As for its polymerization mechanism, it was very complicated. The rapid and smooth polymerization of urushiol was realized by multicomponent synergistic effect in the lacquer sap. The research is helpful to the development of the biomimetic synthesis of lacquer sap and the new green chemical reaction systems with high concentration and viscosity.

## Methods

### Materials

Raw lacquer sap (LS) collected from *R. Vernicifera* lacquer tree was obtained from Lichuan Delong Co., Ltd. (China). It had moisture content of 20.4 wt%. Another lacquer sap was also collected from *R. Vernicifera* and has been stored at room temperature for 4 years. It contained 15.2% of water. Its drying speed was low due to its low laccase activity. It was recorded as LS4. Acetone power, lacquer polysaccharide (LP) and urushiol was extracted from LS based on the process described in the Ref.^23^. Acetone was added into lacquer sap (v/v, 5:1). After stirring for 30 min, the solution was set aside for 5 h. Then, the supernatant was removed. After these steps were repeated three times, the solution was filtered. The precipitation was collected and washed with acetone until the filtrate was colorless. Acetone powder was obtained after drying at room temperature. Lacquer polysaccharide was obtained from acetone powder and urushiol was isolated from the supernatant. Laccase and stellacyanin were isolated and purified from acetone powder according to the method in the literature^17^. Fluorescein isothiocyanate (FITC) was purchased from Wuhan Huashun Biotechnology Co., China. All other chemicals and reagents were used as received.

### Preparation of urushiol with water-insoluble glycoprotein (UGP) and its component analysis

LS was centrifuged (× 16,000g) on an Eppendorf centrifuge 5,418 (Germany). The urushiol with water-insoluble glycoprotein (UGP) was obtained on the upper layer. UGP and the emulsion at the bottom layer were treated by the addition of acetone based on the process described in the Ref.^23^, respectively. Acetone solution was centrifuged and the supernatant was removed. Precipitation was then washed with acetone until the supernatant was colorless. The obtained acetone powers were extracted with water. The absorbance of the aqueous solution at 614 nm was measured. Taking laccase as the standard sample, the approximate content of water-soluble glycoprotein containing copper ion was obtained. At the same time, the insoluble substance after water extraction was freeze-dried to obtain water-insoluble glycoprotein. In addition, raw lacquer sap was also analyzed in the same way.

### Preparation of various urushiol/H_2_O emulsions and their properties

Laccase was dissolved into water and added into urushiol in two different ratios (0.24 wt% and 0.12 wt%). The water in the samples was controlled at 21 wt%. Then the two mixtures were emulsified using a homogenizer under the stirring rate of 12,500 rpm for 2 min. The emulsions were sealed and stored at room temperature for 24 h. In addition, based on the above method, urushiol/H_2_O emulsion, various urushiol/H_2_O emulsions with laccase and stellacyanin, urushiol/H_2_O emulsion with laccase, stellacyanin and lacquer polysaccharide, UGP/H_2_O emulsion with laccase, and UGP/H_2_O emulsion with laccase and stellacyanin were also prepared. The water in all emulsions was controlled at 21 wt%. The stability of these emulsions was observed at room temperature. After they were applied on a glass with a thickness of 50 μm, their drying properties were measured using an automatic drying time tester according to the method in the literature^35^.

### Preparation of ultra-thin sections of the cured lacquer film

The ultra-thin sections of the cured lacquer film were prepared as follows: Firstly, raw lacquer sap was applied on a glass plate with a thickness of 50 μm and dried at 30 °C and 80% relative humidity. Then, the cured lacquer film was immobilized in the embedding plate by epoxy resin AB glue for 24 h. Thereafter, the fixed lacquer film sample was roughly pruned and ground into a taper tip, and was further finished by a Leica EM UC7 ultramicrotome with a magnifying glass. The size dimension of ultra-thin sections was 0.2 mm × 0.05 mm, and the thickness was set at 80 nm with a slicing speed of 1.0 mm s^−1^. Finally, the serial ultra-thin sections of the cured lacquer film were successfully obtained and were observed by a transmission electron microscope (TEM) after phosphotungstic acid (5 wt%) staining.

### Preparation of FITC-labelled laccase

Laccase (24.6 mg) was dissolved into 25 ml of phosphate buffer solution (PBS, pH 7.4). Then FITC (20.2 mg) was added to the laccase solution and the mixture was stirred at room temperature for 13 h. After reaction, the mixture was dialyzed against PBS (pH 7.4) until the absorption value of the solution outside the dialysis tubing was 0 at 495 nm. The dialysate was stored at 4 °C for usage.

### Characterization

The water-drop’s shapes in raw lacquer sap were observed by an Olympus BX43 biological microscope with ToupCam digital camera (Japan). Raw lacquer sap was applied on a glass slide and covered by a cover slide. One drop of cedar oil was placed on the cover slide before the test.

FITC-labelled laccase aqueous solution was added into raw lacquer sap and mixed evenly. The mixture was sealed and kept in the dark at 4 °C for 24 h allowing equilibration. After the mixture was applied on a glass slide, it was observed by a Nikon Eclipse Ti-S inverted fluorescence microscope (Japan) after the excitation with 490–495 nm of light.

Raw lacquer sap was frozen in a device containing liquid nitrogen and then fractured by breaking, and the moisture in the sample was removed through the sublimation at − 110 °C under vacuum. After the sample was coated with gold, the fracture-section morphology of the frozen raw lacquer sap sample was examined using a Hitachi S-4800 field emission scanning electron microscope (FESEM, Japan) equipped with K1250X cryo-stage (Quorum, UK) and elemental microanalysis was performed by a Thermo Noran energy dispersive X-ray spectrometry (EDS, USA). In addition, the surface morphology of the cured lacquer film was also determined using a Zeiss SUPRA55 FESEM (Germany).

A FEI Tecnai G2 F30 S-TWIN field emission transmission electron microscope (FETEM, USA) was used for the characterization of the morphology of the ultra-thin section of the lacquer film and its elemental distribution was analyzed by an EDAX Genesis XM EDS (USA).

Small angle X-ray scattering (SAXS) experiment was performed on a SAXSess mc^2^ SAXS instrument (Anton Paar, Austria) with a slit collimation system using CuKα radiation (wavelength λ = 0.15418 nm) generated by an X-ray generator (40 kV, 50 mA). Before testing, sample was injected into a capillary with a diameter of 1 mm. Test time was 30 min. The experimental data were processed by the following equation for two-phase system^15,16^:

$$q^{{4}} I\left( q \right) \, = {\text{ K}}_{{\text{p}}} \left( {{1 } - \sigma^{{2}} q^{{2}} } \right)$$

where *q* = 4π sin θ/λ, *q* is the scattering vector, *I*(*q*) is the slit-smeared background-corrected scattering intensity, K*p* is a constant, σ is an interface thickness parameter between two phases, 2θ is the scattering angle and λ is the X-ray wavelength.

The viscosity of samples was measured using a Brookfield LVDV-II + Pro viscometer equipped with small sample adapter (USA) at 20 °C.

^1^H NMR spectra were recorded on a Bruker AVANCE III 400 MHz NMR spectrometer (Germany). Samples were prepared according to the method in the literature^35^. In the drying process, samples were taken out and extracted with THF. Then THF was removed by rotary evaporation after filtration, and residues were dissolved in CDCl_3_ for the test.

ESR analysis was carried out at room temperature on a Bruker MicroESR spectrometer (Germany). Samples were coated on the inner surface of the quartz ESR tube and tested under exposure to air.

## Supplementary information

Supplementary Information.

## Data Availability

The data that support the findings of this study are available from the corresponding author on reasonable request.
