# Supplementary material for: Polymerization mechanism of natural lacquer sap with special phase structure
Source: Sci Rep. 2020 Jul 30;10:12867. doi: 10.1038/s41598-020-69823-0 (PMC7393129; doi:10.1038/s41598-020-69823-0)
Supplement: Supplementary file 1 — Supplementary Information. [file 41598_2020_69823_MOESM1_ESM.pdf]

Supplementary Information

Polymerization Mechanism of Natural Lacquer Sap with Special Phase Structure

Jianhong Yang[1,*], Nan Chen[1], Jianfeng Zhu[1], Jun Cai[2], Jianping Deng[1], Feifei Pan[1], Lianghe Gao[1], Zhenfei Jiang[1] & Fengqin Shen[3]

[1]School of Environment and Safety Engineering, Changzhou University, Changzhou 213164, China

[2]Key Laboratory of Fermentation Engineering (Ministry of Education), Hubei Key Laboratory of Industrial Microbiology, Hubei University of Technology, Wuhan 430068, China.

[3]Changzhou Liu Guojun Vocational Technology College, Changzhou 213025, Jiangsu, China

*Correspondence to: yangjianhong@cczu.edu.cn

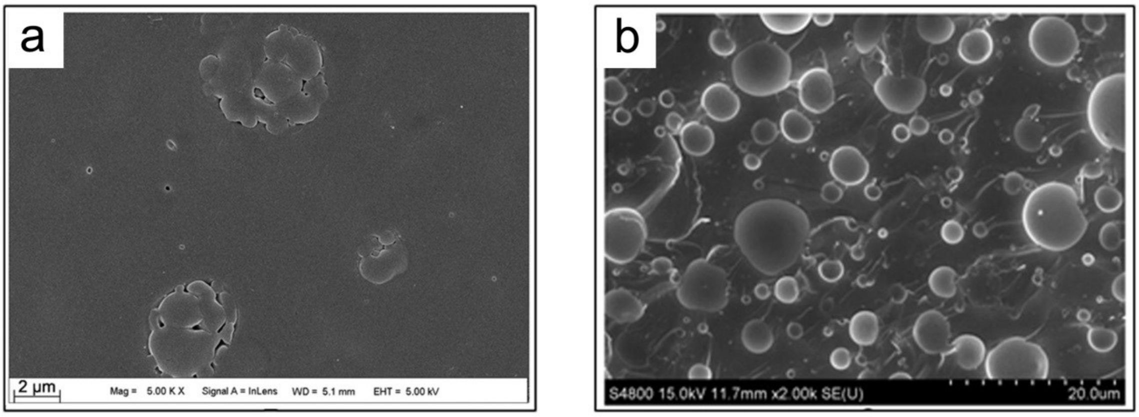

Supplementary Fig. S1. SEM image of the cured lacquer film surface (a) and Cryo-FESEM images of the frozen lacquer sap (b).

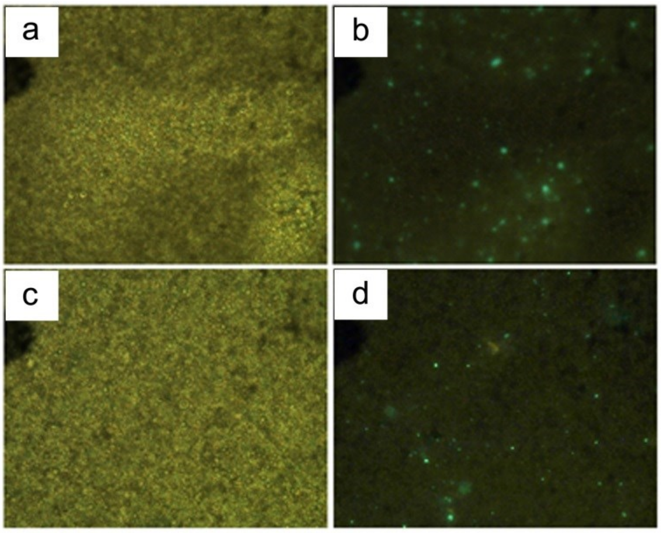

Supplementary Fig. S2. The distribution of fluorescence-labelled laccase in the raw lacquer sap. The results were obtained from an inverted fluorescence microscope. a and c are the bright field images of b and d, respectively.

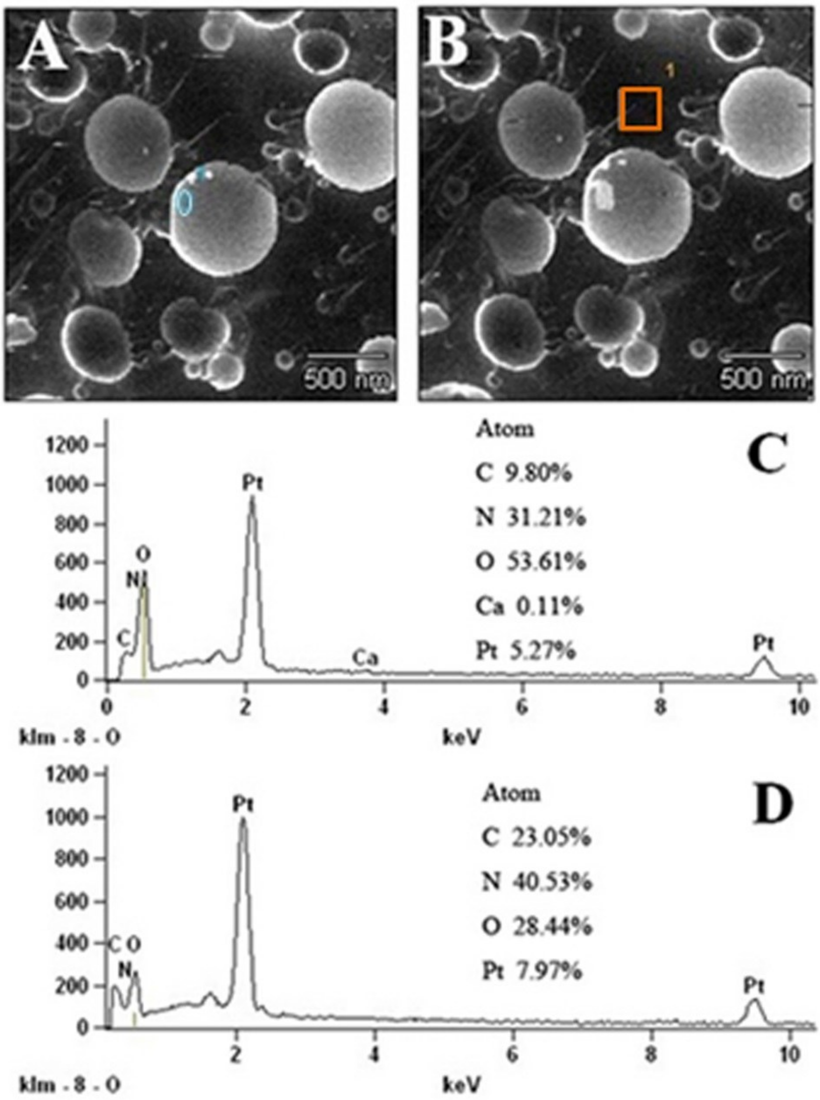

Supplementary Fig. S3. Cryo-FESEM-EDS analysis on the frozen fracture surface of raw lacquer sap. (c) EDS spectrum of the selected area in a (blue circle); (d) EDS spectrum of the selected area in b (brown square).

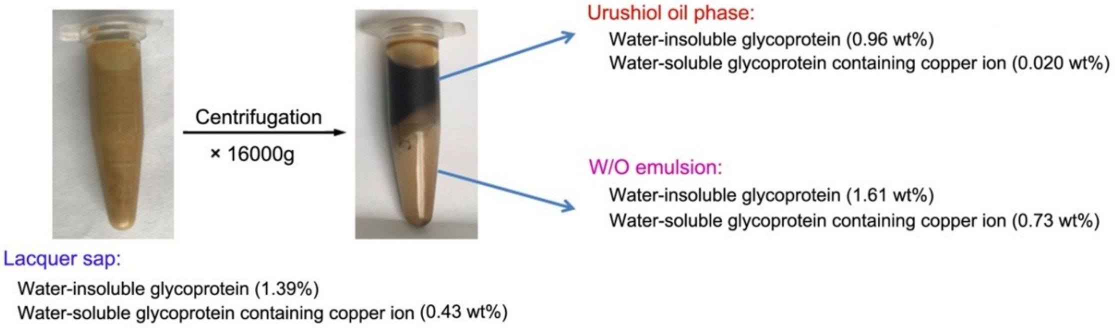

Supplementary Fig. S4. Preparation of urushiol with water-insoluble glycoprotein (UGP) and its components.

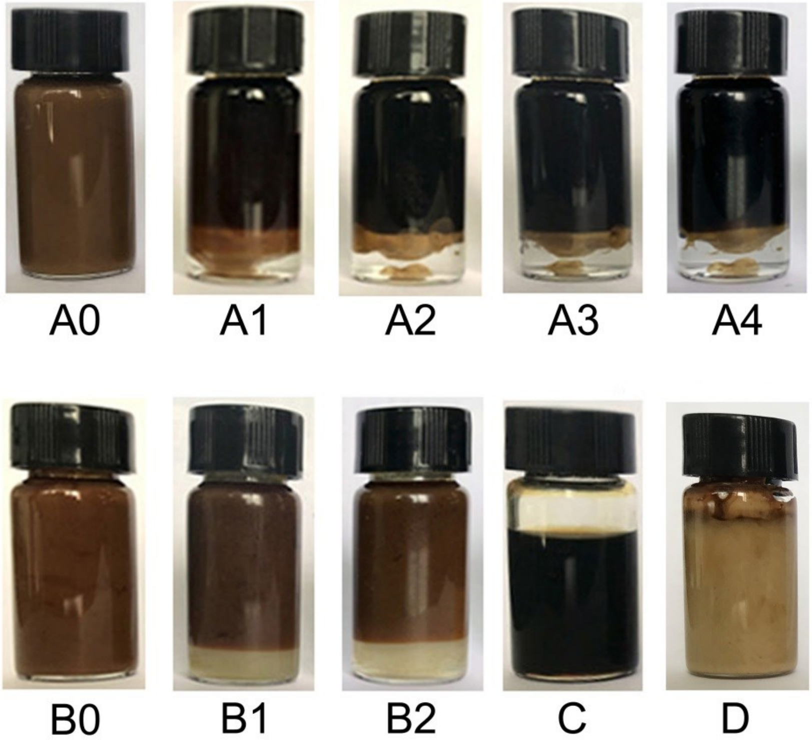

Supplementary Fig. S5. The stability of the urushiol/H_2O emulsion and urushiol/H_2O emulsion with laccase, lacquer polysaccharide and stellacyanin. (A) Urushiol/H_2O emulsion (21 wt% of water). Storage period: A0, 0 h; A1, 24 h; A2, 72 h; A3, 96 h; A4, 144 h. (B) Urushiol/H_2O emulsion with 0.12wt% of laccase, 4.0 wt% of lacquer polysaccharide and 0.012 wt% of stellacyanin. Storage period: B0, 0 h; B1, 24 h; B2, 72 h. (C) Urushiol; (D) Raw lacquer sap. It can be stored steadily for several years.

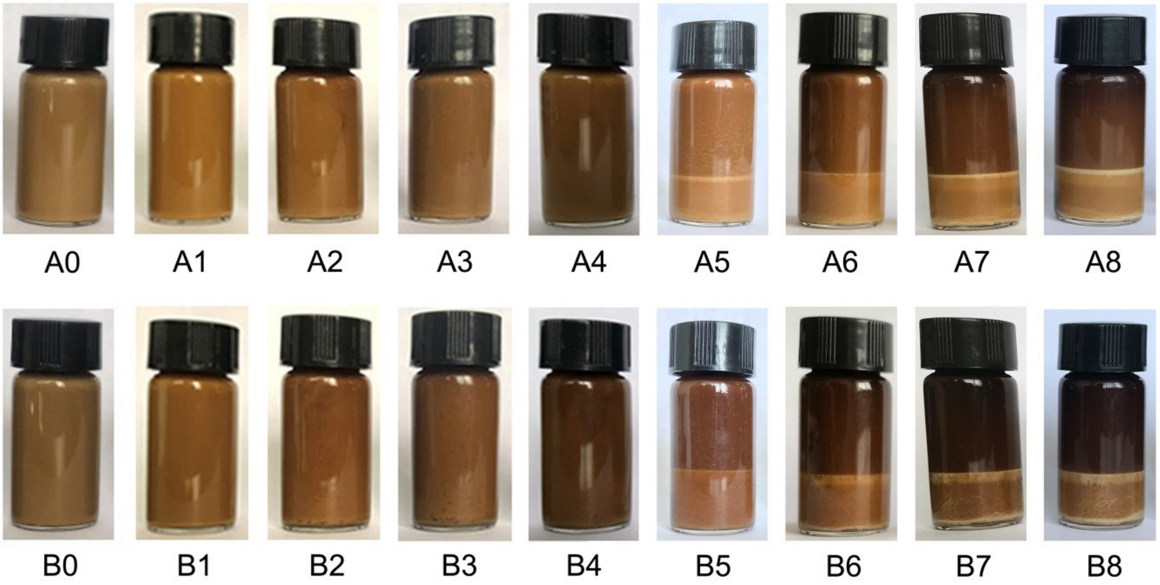

Supplementary Fig. S6. The stability of the urushiol/H_2O emulsion with laccase (UHEL). (A) Urushiol/H_2O emulsion with 21 wt% of water and 0.24 wt% of laccase. Storage period: A0, 0 h; A1, 24 h; A2, 72 h; A3, 96 h; A4, 144 h; A5, 12 days; A6, 28 days; A7, 57 days; A8, 76 days.(B) Urushiol/H_2O emulsion with 21 wt% of water and 0.12 wt% of laccase. Storage period: B0, 0 h; B1, 24 h; B2, 72 h; B3, 96 h; B4 ,144 h; B5, 12 days; B6, 28 days; B7, 57 days; B8, 76 days.

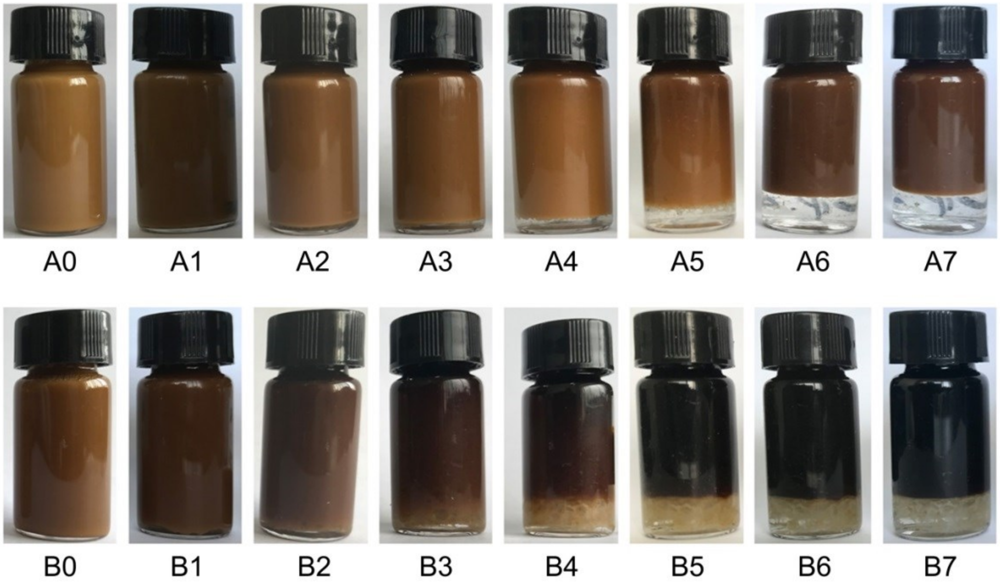

Supplementary Fig. S7. The stability of the urushiol/H_2O emulsions with stellacyanin. (A) Urushiol/H_2O emulsion with 21 wt% of water and 0.12 wt% of stellacyanin. Storage period: A0, 0 h; A1, 24 h; A2, 48 h; A3, 72 h; A4, 5 days; A5, 9 days; A6, 29 days; A7, 48 days; (B) Urushiol/H_2O emulsion with 21 wt% of water and 0.012 wt% of stellacyanin. Storage period: B0, 0 h; B1, 24 h; B2, 48 h; B3, 72 h; B4, 5 days; B5, 9 days; B6, 29 days; B7, 48 days.

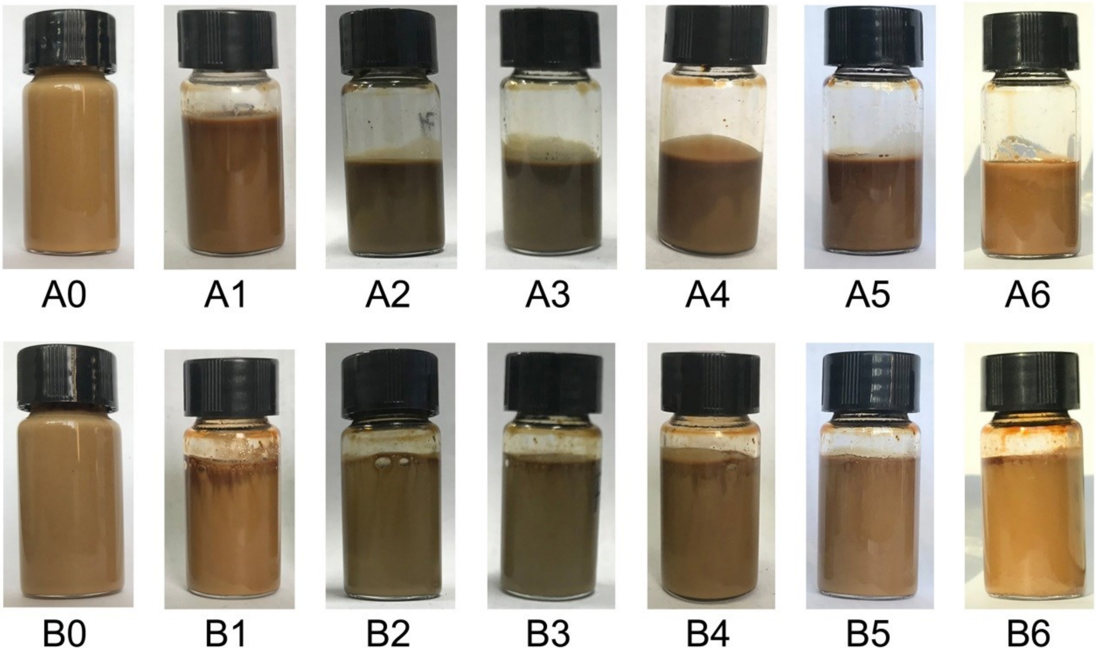

Supplementary Fig. S8. The stability of the UGP/H_2O emulsion and UGP/H_2O emulsion with laccase. (A) UGP/H_2O emulsion with 21 wt% of water. Storage period: A0, 0 days; A1, 10 days; A2, 15 days; A3, 22 days; A4, 36 days; A5, 45 days; A6, 113 days; (B) UGP/H_2O emulsion with 21 wt% of water and 0.24 wt% of laccase (UGPHL). Storage period: B0, 0 days; B1, 10 days; B2, 15 days; B3, 22 days; B4, 36 days; B5, 45 days; B6, 113 days. UGP contained 0.96 wt% of water-insoluble glycoprotein (WIGP).

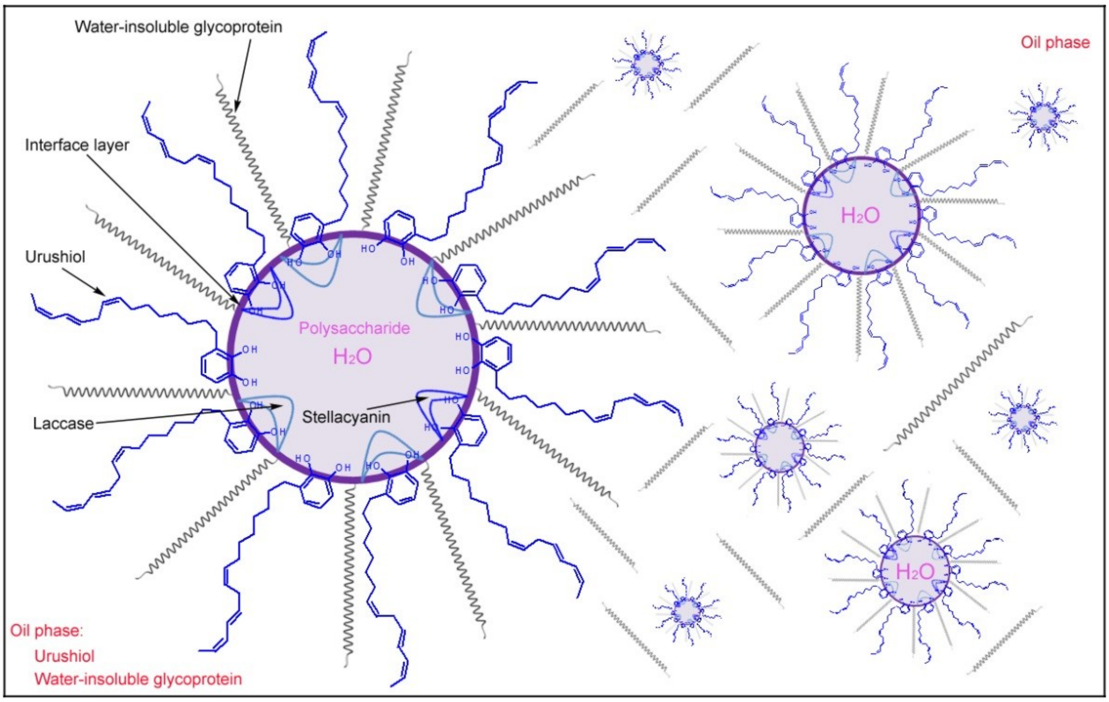

Supplementary Fig. S9. The possible phase structure of raw lacquer sap. There are lacquer polysaccharide, laccase and stellacyanin in aqueous phase and water-insoluble glycoprotein in oil phase and the interface layer. The phase interface layer is composed of urushiol, laccase-urushiol complex, stellacyanin-urushiol complex and water-insoluble glycoprotein.

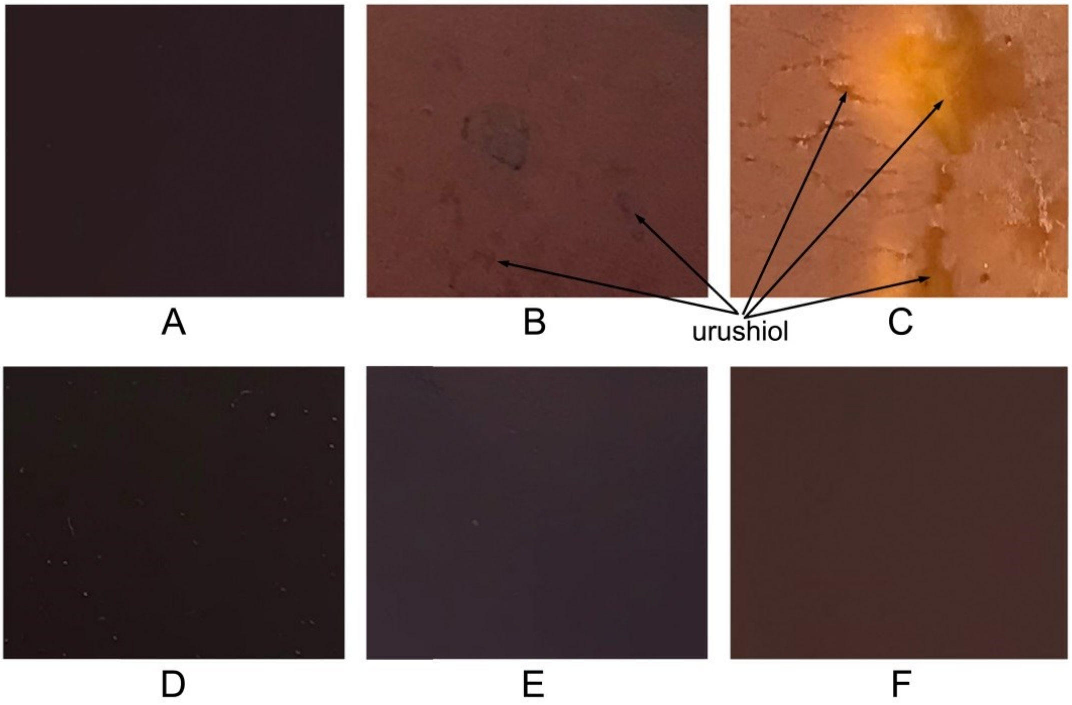

Supplementary Fig. S10. The photos of lacquer films. (A) lacquer sap (LS) ; (B) urushiol/H_2O emulsion with 21 wt% of water and 0.24 wt% of laccase; (C) urushiol/H_2O emulsion with 21 wt% of water and 0.12 wt% of laccase; (D) lacquer sap (LS4) with 0.24 wt% of active laccase; (E) UGP/H_2O emulsion with 21 wt% of water and 0.24 wt% of laccase; (F) UGP/H_2O emulsion with 21 wt% of water and 0.12 wt% of laccase.

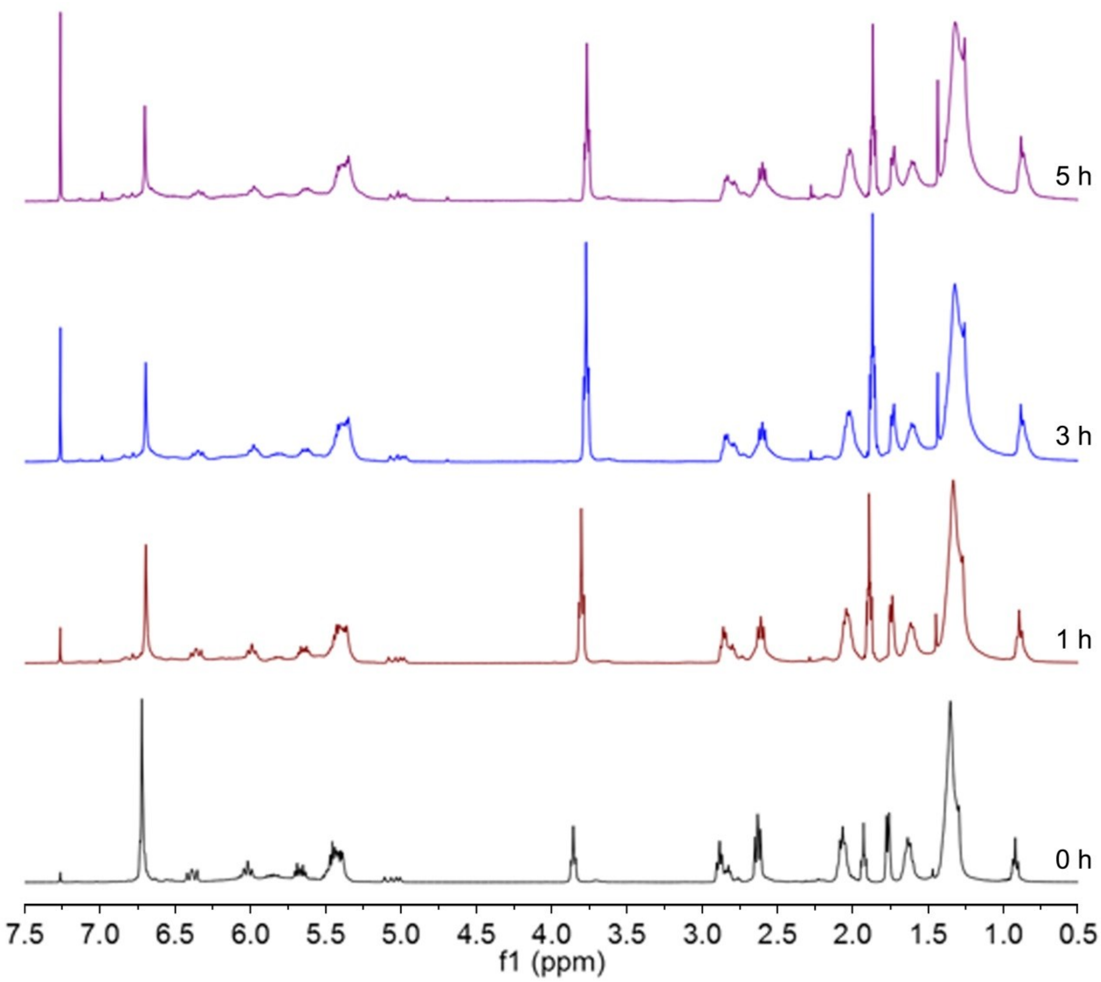

Supplementary Fig. S11. ^{1}H NMR spectrum of THF extract after UGP/H_2O emulsion with 0.24 wt% of laccase (UGPHL) drying for 0 h, 1h , 3h and 5h.

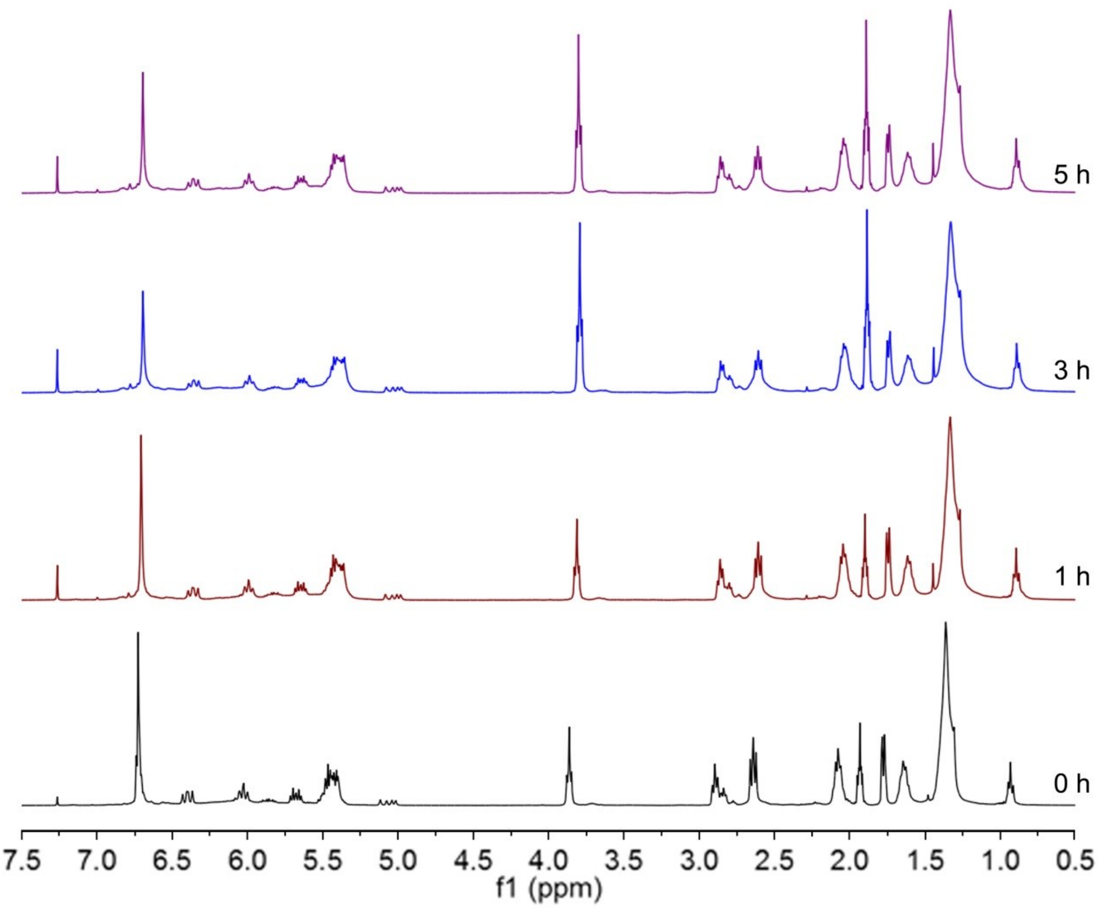

Supplementary Fig. S12. ^{1}H NMR spectrum of THF extract after UGP/H_2O emulsion with 0.24% of laccase and 0.024 wt% stellacyanin (UGPHL-S) drying for 0 h, 1h, 3h and 5h.

Supplementary Table S1

The change of H atom content on benzene ring during the drying of lacquer sap, urushiol/H_2O emulsion and UGP/H_2O emulsion.

Sample[a]	Additive 1	Ratio (wt%)	Additive 2	Ratio (wt%)	Temp. (^oC)	RH (%)	Decrease of H atom on phenyl ring (%)[b]		
							1 h	3 h	5 h
LS	-	-	-	-	30	80	32.0	56.0	-
UHEL	Laccase	0.24	-		30	80	32.5	47.1	47.4
UHEL-S	Laccase	0.24	Stellacyanin	0.024	30	80	17.6	18.1	19.6
UGPHL	Laccase	0.24	-	-	30	80	42.7	59.2	67.1
UGPHL-S	Laccase	0.24	Stellacyanin	0.024	30	80	30.3	47.2	46.4

[a]LS: lacquer sap; UHEL: urushiol/H_2O emulsion with active laccase; UHEL-S: urushiol/H_2O emulsion with active laccase and stellacyanin; UGPHL: UGP/H_2O emulsion with active laccase; UGPHL-S: UGP/H_2O emulsion with active laccase and stellacyanin. [b]The results were obtained from the corresponding ^1H NMR spectra.
